# Comparison of Thermal and Non-Thermal Processing of Swine Feed and the Use of Selected Feed Additives on Inactivation of Porcine Epidemic Diarrhea Virus (PEDV)

**DOI:** 10.1371/journal.pone.0158128

**Published:** 2016-06-24

**Authors:** Michaela P. Trudeau, Harsha Verma, Fernando Sampedro, Pedro E. Urriola, Gerald C. Shurson, Jessica McKelvey, Suresh D. Pillai, Sagar M. Goyal

**Affiliations:** 1 Department of Animal Science, University of Minnesota, St. Paul, Minnesota, 55018, United States of America; 2 Department of Veterinary Population Medicine University of Minnesota, St. Paul, Minnesota, 55018, United States of America; 3 Center for Animal Health and Food Safety, University of Minnesota, St. Paul, Minnesota, 55018, United States of America; 4 National Center for Electron Beam Research and IAEA Collaborating Centre for Electron Beam Technology,Texas A&M University, College Station, Texas, 77843, United States of America; Sun Yat-sen University, CHINA

## Abstract

Infection with porcine epidemic diarrhea virus (PEDV) causes diarrhea, vomiting, and high mortality in suckling pigs. Contaminated feed has been suggested as a vehicle of transmission for PEDV. The objective of this study was to compare thermal and electron beam processing, and the inclusion of feed additives on the inactivation of PEDV in feed. Feed samples were spiked with PEDV and then heated to 120–145°C for up to 30 min or irradiated at 0–50 kGy. Another set of feed samples spiked with PEDV and mixed with Ultracid P (Nutriad), Activate DA (Novus International), KEM-GEST (Kemin Agrifood), Acid Booster (Agri-Nutrition), sugar or salt was incubated at room temperature (~25°C) for up to 21 days. At the end of incubation, the virus titers were determined by inoculation of Vero-81 cells and the virus inactivation kinetics were modeled using the Weibull distribution model. The Weibull kinetic parameter delta represented the time or eBeam dose required to reduce virus concentration by 1 log. For thermal processing, delta values ranged from 16.52 min at 120°C to 1.30 min at 145°C. For eBeam processing, a target dose of 50 kGy reduced PEDV concentration by 3 log. All additives tested were effective in reducing the survival of PEDV when compared with the control sample (delta = 17.23 days). Activate DA (0.81) and KEM-GEST (3.28) produced the fastest inactivation. In conclusion, heating swine feed at temperatures over 130°C or eBeam processing of feed with a dose over 50 kGy are effective processing steps to reduce PEDV survival. Additionally, the inclusion of selected additives can decrease PEDV survivability.

## Introduction

Porcine Epidemic Diarrhea Virus (PEDV) is a pleomorphic, enveloped RNA virus, classified as a coronavirus under the family *Coronaviridae* [1]. The virus was first identified during an outbreak of diarrhea on a Belgian swine breeding farm in 1978 [2]. Upon infection with PEDV, suckling pigs experience diarrhea, vomiting, and high mortality [3]. Since the initial identification of PEDV, it has been reported in Canada, Korea, China, Thailand, Italy, Hungary, and Vietnam [4]. It is important to note that China and Vietnam are two of the top swine producing countries in Asia and that they were both impacted by PEDV [5].

In the United States, the virus was first detected in April 2013 and has since caused high piglet mortality in over 17 states [6]. Some have suggested that PEDV is transmitted via contaminated feed [7]. Although research has been conducted to show the survival of PEDV in feed and feed ingredients [8–10] there is limited data comparing feed processing treatments and their efficacy in inactivating the virus in complete swine feed.

In the feed and ingredient industry, temperature and time conditions vary with the processing method used e.g., pelleting, rendering, pasteurization, or spray drying. Moisture content of feed and ingredients is also impacted by the type of processing method used. The interactions between temperature, time, and moisture play a role in virus inactivation in feed. Ingredients of porcine origin have been thought to have the greatest risk for disease transmission [9]. When processing rendered ingredients for animal feed, the processing conditions vary based on the composition of the raw material. In general, the National Renderers Association suggests temperatures between 115°C and 145°C for 40 to 90 min for most rendering systems. Such temperatures are deemed effective in inactivating pathogens such as Salmonella spp. [11]. Similarly, the enveloped classical swine fever RNA virus is readily inactivated in pasteurization processes after only 1 min at 71°C [12]. High temperatures (around 100°C) are also used in other processes including micronization to prepare cereal grains [13,14]. The high temperatures used in processing rendered products and cereal grains may also be effective in inactivating PEDV in contaminated feed, but no research has been conducted to compare PEDV survival at temperatures greater than 80°C.

Other processing procedures such as ionizing irradiation have been shown to reduce the survivability of pathogens in feed, specifically *Listeria monocytogenes* in poultry feed [15]. More recent applications of ionizing radiation technology such as electron beam (eBeam) are now used to routinely pasteurize foods and decontaminate animal feeds from pathogens [16,17]. The main characteristics of eBeam technology include its non-thermal nature (thereby reducing potential nutrient losses), utilization of commercial electricity (does not rely on radioactive isotopes), high speed processing, and the ability to precisely control the dose delivered [17,18]. The US Food and Drug Administration (FDA) has recognized this process as a treatment method for food and animal feed for doses up to 50 kGy [19]. All of these attributes make the eBeam technology attractive for a wide variety of applications, including pasteurization of animal feed.

In addition to thermal and eBeam treatments, certain organic acid feed additives including propionic, formic, and butyric acid have been used in the past for their antimicrobial properties. These additives have previously been used as a method of controlling pathogens such as Salmonella spp. and *Escherichia coli* in poultry feed and other matrices [20,21]. However, the effects of organic acids and other additives including sugar and salt in feed have not been investigated. Therefore, the objective of this study was to determine if thermal and non-thermal methods of microbial inactivation, as well as the use of selected feed additives, are effective in reducing the survival of PEDV in experimentally contaminated swine feed.

## Materials and Methods

### Virus and virus recovery

The NVSL (National Veterinary Service Laboratory, Ames, IA) strain of PEDV was propagated and titrated in Vero-81 (African Green monkey kidney, ATCC^®^ CCL-81^™^) cells. We have found that both Vero-76 and Vero-81 cells are susceptible to PEDV. We chose to use vero-81 in this study. The cells were grown in Dulbecco's Modified Eagle Medium (DMEM; Mediatech, Herndon, VA, USA) containing 8% fetal bovine serum (FBS; Gibco, Life Technologies, Grand Island, NY, USA), 50 μg/mL gentamicin (Mediatech), 150 μg/mL neomycin sulfate (Sigma, St. Louis, MO, USA), 1.5 μg/mL fungizone (Sigma), and 455 μg/mL streptomycin (Sigma). The cells were washed three times with phosphate buffered saline (PBS; pH 7.2). After virus inoculation, the cells were incubated at 37°C for 1 h, allowing virus adsorption using maintenance medium (DMEM with gentamicin, neomycin sulfate, fungizone, streptomycin and 10 μg/mL trypsin). The cells were washed again 60 minutes after inoculation. The washing medium was Hanks' balanced salt solution containing no trypsin. However, the maintenance medium did contain trypsin. Inoculated cells were incubated at 37°C under 5% CO_2_. Cells were examined daily under an inverted microscope for the appearance of virus-induced cytopathic effects (CPE) for up to five days post-infection. The virus was harvested by subjecting the infected cells to three freeze-thaw cycles (-80°C/25°C) followed by centrifugation at 2500 × g for 15 min at 4°C. The supernatant was collected and aliquoted into 50 mL tubes followed by storage at -80°C until used.

In all experiments, the surviving virus after a certain treatment was recovered in an eluent consisting of a 3% solution of beef extract solution (Lab Scientific, Highlands, NJ) in 0.05M glycine (Sigma, St. Louis, MO), pH 7.5. Following elution, the eluate was centrifuged for 10 min at 2500 x g to remove organic matter/debris. The supernatants were used to determine the amount of surviving virus, if any. For titration of PEDV in virus stock and other samples, serial ten-fold dilutions of the eluates prepared in DMEM (maintenance medium) were inoculated into Vero-81 monolayers contained in 96-well microtiter plates (Nunc, NY, USA) using 100 μL/well and three wells per dilution. Inoculated cells were incubated at 37°C under 5% CO_2_ for up to seven days until the CPE appeared. The highest dilution showing CPE was considered the end point. Virus titers were calculated as a median tissue culture infectious dose (TCID_50_/mL) by the Karber method [22]. The amount of surviving virus was compared with the starting virus titer to calculate the amount of inactivated virus and was expressed in log scale (log_10_ TCID_50_/mL).

### Inactivation of PEDV by thermal treatment

A complete phase II pig starter feed (CGI, enhanced NP-NT, batch no. 831458) was obtained and confirmed to be PEDV negative by RT-PCR. The proximate analysis of this feed sample is displayed in Table 1. Aliquots of feed (5 g amounts) were prepared in glass beakers, which were then placed into drying ovens set at 120, 130, 140, and 145°C. The feed aliquots remained in the ovens for 30 min to reach oven temperature. Once the feed reached the indicated temperature, the beakers were immediately removed and spiked with 1 mL of PEDV (6.8x10^3^ TCID_50_/mL) that was previously tempered at room temperature (~25°C). After mixing, beakers were placed back in the ovens at the appropriate temperatures and incubated for 0, 5, 10, 15, 20, 25, and 30 min. After the incubation period, the samples were removed from the oven, actively cooled with a fan for 15 min, the virus eluted from the feed using the 3% beef extract eluent solution, and the number of surviving viral particles titrated. Triplicate samples of each temperature were combined for a single titration and inoculation into cells. The experiment was performed in duplicate.

**Table 1:** Proximate Analysis of the Vita-Plus CGI enhanced feed used for the experiment.

Analysis	Percent (%)
Moisture	8.57
Protein	24.2
Fat	4.47
Fiber	2.02
Ash	9.45

### Inactivation of PEDV by eBeam processing

Preliminary dose mapping experiments were performed to calculate the dose uniformity within the samples. A dose-uniformity ratio was calculated by maximum dose/minimum dose for each experiment. Under ideal circumstances, the dose-uniformity ratio (DUR) should be about 1.0. During commercial processing of animal feed using eBeam technology, the size of the final package needs to be optimized to assure dose uniformity. The optimization involves adjusting the dimensions and weight to ensure adequate penetration of the eBeam electrons as well as dose uniformity within the package. Under commercial processing conditions, the goal is to try to attain as uniform a dose as possible. At the eBeam facility on the TAMU campus, the DUR of a commercial pet food product is 1.78. Aliquots of complete phase II pig starter feed (5 g amounts) were prepared in several 50 mL plastic centrifuge tubes followed by the addition of 1 mL of PEDV to each tube. After mixing, the virus-spiked feed was placed into individual plastic bags. The bags were flattened to remove all of the air and create a thin, even layer of the feed sample. The bags were triple-sealed to meet the biosafety procedures at the eBeam irradiation facility. The sealed bags were shipped on ice via overnight shipping to the National Center for Electron Beam Research at Texas A&M University, College Station, TX.

Preliminary dose delivery trials were performed to determine the appropriate conveyor speed and other specifications to achieve the target doses. On the day of arrival, the samples were exposed to target eBeam doses of 10, 20, 30, and 50 kGy. A control sample was also shipped with the irradiated samples, but it was not exposed to eBeam irradiation at the facility. After treatment, the samples (including the controls) were shipped back to the University of Minnesota (St. Paul) on ice via overnight shipping. Upon arrival, the samples were immediately eluted using the aforesaid eluent solution. This experiment was performed only once.

### Effect of feed additives on PEDV survivability in feed

Aliquots of feed in 5 g amounts were placed in plastic scintillation vials. The amount of feed additive used in the 5 g sample was determined based on the recommended dosage from the manufacturers. The following additives were added to separate aliquots of feed: 0.015 g Ultracid P (Nutriad, Dendermonde, Belgium), 0.02 g Activate DA (Novus International, St. Charles, MO), 0.01 g of Acid Booster (Agri-Nutrition, DeForest, WI), 0.01 g KEM-GEST (Kemin Agrifoods, Des Moines, IA), 0.02 g of granulated sugar (Shoppers Value, Eden Prairie, MN) and 0.02 g of commercial salt (Essential Everyday, Eden Prairie, MN). Another set of vials was used as paired controls without the addition of any feed additive. After the additives were added to the appropriate vials, 1 mL of PEDV was added to each vial followed by vortexing. All vials were incubated at room temperature (around 25°C during the season which this experiment was performed). After incubation for 0, 1, 3, 5, 7, 14, and 21 days, the surviving virus was eluted from the samples using the 3% beef extract eluent solution. The experiment was performed in duplicate. Each experiment used a set of triplicate samples that were combined and used for a single titration and inoculation into cells.

After completion of the trial, the pH of each feed sample solution with and without the additive was measured. The pH was measured by adding 50 mL of deionized water to 5 g of feed followed by mixing continuously on a magnetic stirrer for 15 min at room temperature. After mixing, a pH probe was used to measure the pH of the liquid. The pH measurement experiment was performed in triplicate. In addition to measuring pH, the active ingredients for Activate DA (2-hydroxy-4-methylthiobutanoic acid), KEM-GEST (phosphoric, fumaric, lactic, and citric acids), sugar (sucrose), Acid Booster (phosphoric, citric, and lactic acids), salt (sodium chloride), and Ultracid P (orthophosphoric, citric, fumaric, and malic acids) were compared.

### Statistical analysis

Inactivation kinetic data (log TCID_50_/mL) were analyzed by GInaFiT, a freeware Add-in for Microsoft Excel developed by Geeraerd et al. [23]. The traditional log-linear model that assumes a linear relationship between the virus concentration and processing time was developed by Bigelow and Esty [24] and used to characterize the survival curves of PEDV by using the following equation:

$$LogN=LogN_{o}-k\times t$$ (1)

where *N* is the surviving virus titer after the treatment (expressed as TCID_50_/mL), *N*_*0*_ is the initial virus titer (TCID_50_/mL), *k* is the kinetic parameter (min^-1^ or day^-1^), and *t* is the treatment time (min or days). The kinetic parameter *k* is usually expressed as *D*, also known as the decimal reduction time (time required at a certain temperature to reduce 90% or 1 log of the initial virus titer) and was calculated as follows:

$$D=\frac{2.3}{K}$$ (2)

The Weibull distribution function has been used to describe non-linear inactivation patterns of various microorganisms after different thermal and non-thermal processing [25]. Assuming that the temperature resistance of the virus is governed by a Weibull distribution, Mafart et al. developed the following Weibullian equation:

$$Log\left(N\right)=Log\left(N_{o}\right)-{\left(\frac{t}{\delta }\right)}^{n}$$ (3)

where *N* is the surviving virus after the treatment expressed as (TCID_50_/mL), *N*_*0*_ is the initial virus titer (TCID_50_/mL), *δ* is the time of the first logarithm decline for the virus titer population (min or days), and *n* is the shape parameter. The *n* value provided a general description of the form of the curve; if n > 1, the curve is convex (it forms shoulders), if n < 1, the curve is concave (it forms tails), and if n = 1, the curve is a straight line and can be described by a linear model.

Two valid replicates were used to evaluate how well the model fit with the experimental data by calculating the adjusted R^2^ (Adj. R^2^) as follows:

$$Adj.R^{2}=\left[1-\frac{\left(m-1\right)(1-\frac{SSQ\;regression}{SSQ\;total}}{m-j}\right]$$ (4)

where m is the number of observations, j is the number of model parameters, and SSQ is the sum of squares.

An ANOVA test using the mixed procedure of SAS (SAS Inst., Cary, NC) was used to determine statistical differences across treatments. Least squared means with Tukey adjustment was used to determine differences between treatment means, with P < 0.05 considered to be significantly different. The experimental unit was the value obtained from the combined triplicate vials. The fixed effects were temperature or feed additive used.

## Results

### Thermal inactivation of PEDV

**Fig 1:**
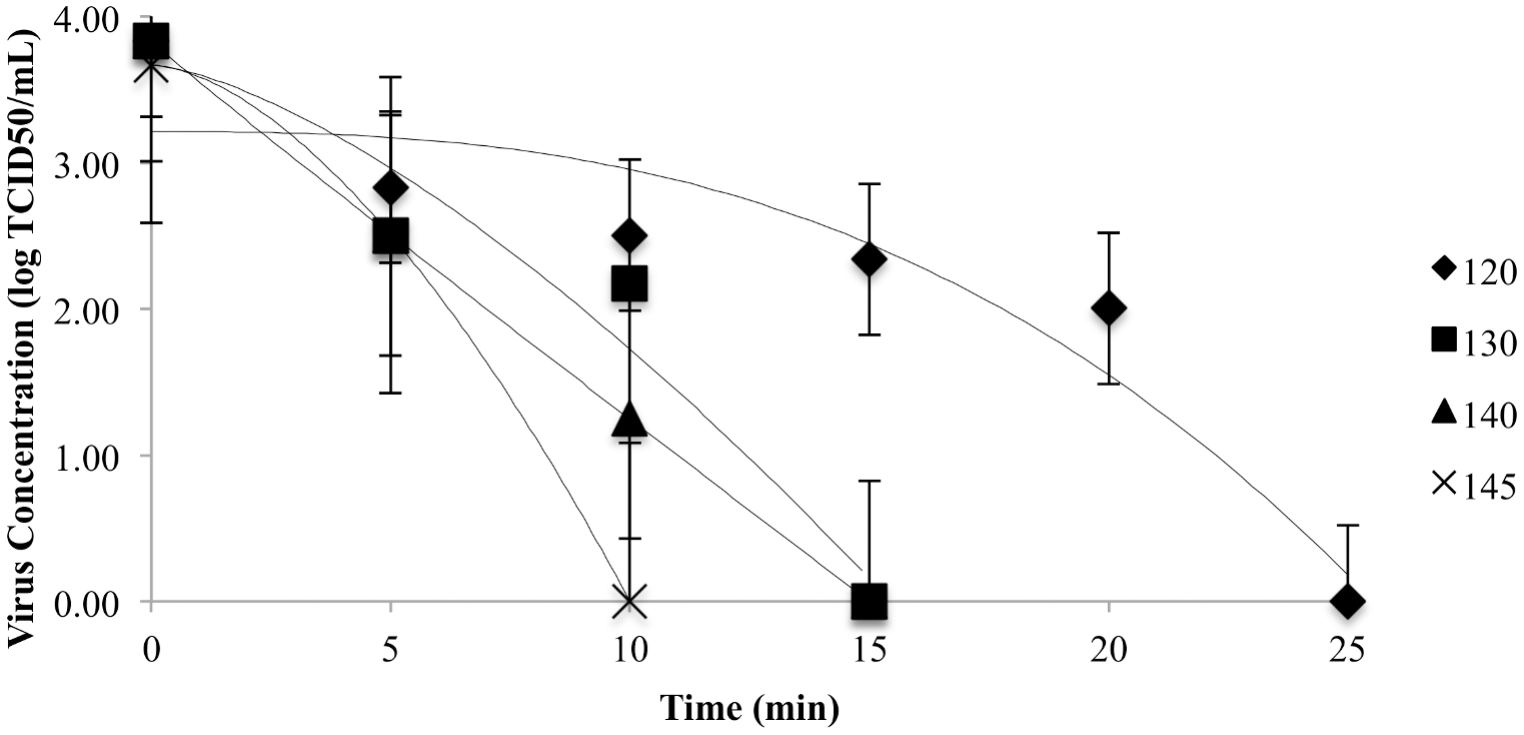
Inactivation of PEDV in complete feed when exposed to thermal processing. The inactivation curves determined by the Weibull model for the survival of PEDV in complete feed at 120°C, 130°C, 140°C, and 145°C.

Virus concentrations (log TCID_50_/mL) at 120, 130, 140, and 145°C as a function of time are shown in Fig 1. Survival curves displayed non-linear behavior (shoulder), meaning a certain exposure time was needed to be surpassed to initiate viral inactivation. The kinetic parameters of the log-linear and Weibull model (D and delta values) for each temperature are shown in Table 2. The PEDV showed a high thermal resistance in the dry feed samples and it was completely inactivated (3.0 log reduction) at each of the tested temperatures within 30 min. The original moisture content of the feed sample was 8.57% (W/V). After adding 1 mL of virus media the moisture increased to 23.8% (W/V). This change in moisture content potentially increased the ability of heat to reduce virus concentration, as heat inactivation is more efficient in higher moisture content as previously demonstrated by the higher sensitivity of bovine parvovirus to moist heat vs. dry heat [26].

**Table 2:** Comparison of Log Linear and Weibull kinetic parameters and goodness of fit for PEDV thermal inactivation kinetics in feed.

	Weibull model			Log Linear model	
Temp	Delta^1^ (min)	Shape Parameter^2^ (n)	Adj. R^2^	D^1^ (min)	Adj. R^2^
120°C	16.52^a^ ±0.08	2.7	0.79	8.07^a^ ± 0.20	0.80
130°C	2.85^b^ ± 0.00	0.7	0.84	6.05^b^ ± 0.00	0.84
140°C	2.10^b^ ± 2.39	0.6	0.82	6.26^b^ ± 0.72	0.73
145°C	1.30^b^ ± 0.88	0.5	0.76	6.77^b^ ± 0.28	0.64

^1^ Different letters within the same column differ (*P* < 0.05)

^2^The shape parameter (n) indicates the shape of the curve with a value n > 1 forming shoulders and being convex, n < 1 forming tails and being concave, and n = 1 being linear.

The Weibull model provided a better fit for the experimental data by generating greater adj. R^2^ values (0.76 to 0.84) than the log-linear model (0.64 to 0.84). Delta values were reduced as the temperature increased indicating a greater inactivation rate. In order to achieve a 5 log virus reduction, predicted exposure times of 82.6, 14.2, 10.5 and 6.5 min were estimated at 120, 130, 140 and 145°C, respectively. Shape parameter (n) was greater than 1 for all temperatures except 120°C, indicating the shoulder appearance of the inactivation curves shown in Fig 1. Delta values at 130, 140, and 145°C were not statistically different (*P* > 0.05).

### Inactivation of PEDV by eBeam processing

**Fig 2:**
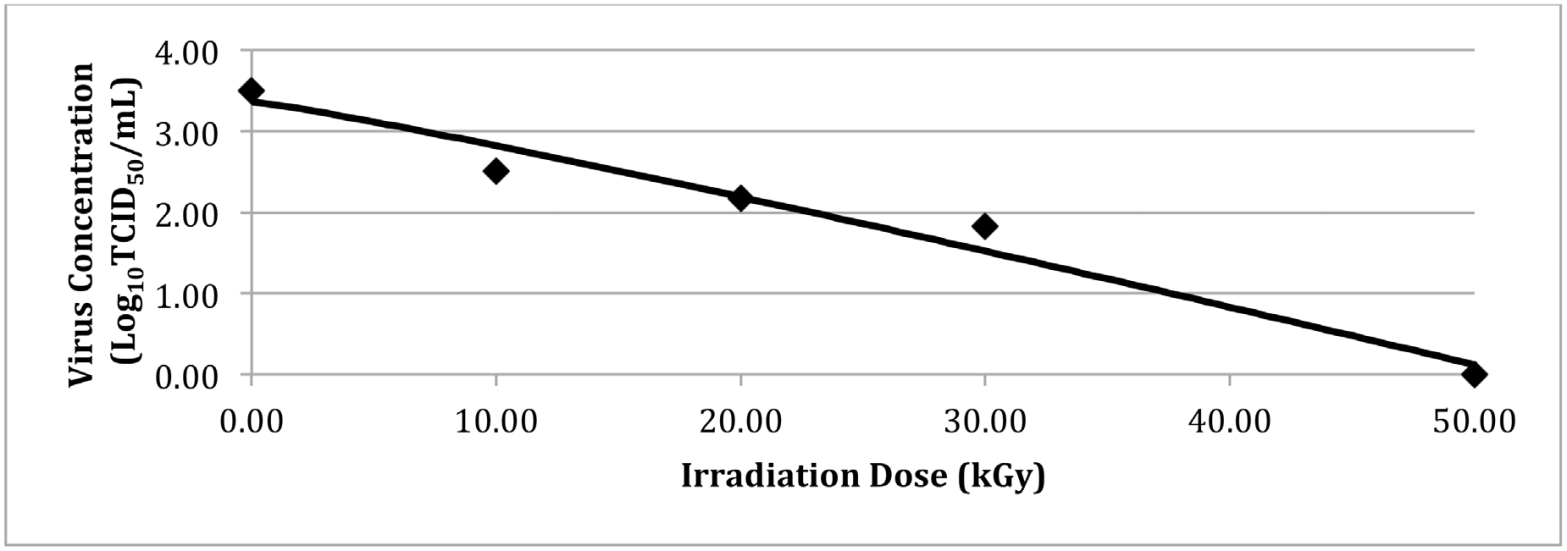
Inactivation of PEDV by eBeam irradiation processing. Inactivation curve modeled by the Weibull model for the survival of PEDV in complete feed when exposed to eBeam irradiation.

Fig 2 shows the results of PEDV inactivation after eBeam processing. The figure represents the inactivation curve predicted by the Weibull model and the points illustrate the observed values. The delta value using the Weibull model was estimated as 17.25 kGy. The survival of PEDV was dose dependent with higher inactivation being achieved with increasing doses. In order to obtain a 5 log reduction of PEDV in feed, a dose of 86.25 KGy would be needed.

### Effect of feed additives on PEDV inactivation

The virus concentration in feed (control sample) and feed with additives is shown in Table 3. The addition of the additives to the feed sample decreased the initial virus titer. The D and delta values of the log-linear and Weibull models respectively, are shown in Table 4. The use of the Weibull model resulted in greater adj. R^2^ values than the log-linear model, and therefore, was used to characterize the survival of PEDV with the addition of additives during storage at room temperature. Delta values for all the additive-containing samples, except Ultracid P and salt, were significantly lower than the control sample (P < 0.05) indicating faster inactivation kinetics (Table 4). After 21 days of incubation at room temperature (25°C), a 3 log reduction was observed in samples containing KEM-GEST, sugar, and salt. In the same 21-day incubation period, a 2 log reduction was observed in Activate DA, and <2 log reduction was observed in the control, Ultracid P, and Acid Booster.

**Table 3:** Comparison of the concentration of PEDV in feed with and without feed additives.

Incubation Period (days)	Average Virus Titer (TCID_50_/mL)						
	Control	Ultracid P	Activate DA	Acid Booster	KEM-GEST	Sugar	Salt
0	5000	3250	6800	3250	5000	6800	3250
1	3460	6800	1500	1500	6800	6800	6800
3	4200	680	150	680	6800	1500	3200
5	8000	680	374	320	320	320	3200
7	4460	680	68	680	68	150	68
14	909	320	68	68	68	320	320
21	252	68	68	68	1	1	1

**Table 4:** Comparison of Log Linear and Weibull kinetic parameters and goodness of fit for PEDV survival kinetics in feed additives.

		Weibull model			Log linear model	
Additive	Log reduction at 21d	Delta^1^ value (days)	Shape Parameter^2^(n)	Adj. R^2^	D value (days)	Adj. R^2^
Control	1.4	17.23^bc^ ± 0.78	1.9	0.83	18.34± 10.01	0.79
Ultracid P	1.6	13.00^ac^ ± 3.41	0.7	0.87	14.90 ± 3.95	0.86
Activate DA	2.0	0.81^a^ ± 0.52	0.2	0.78	13.59± 1.13	0.39
Acid Booster	1.6	7.24^a^ ± 3.71	0.5	0.90	13.25 ± 1.60	0.82
KEM-GEST	3.8	3.28^a^ ± 2.05	0.8	0.83	5.68 ± 0.10	0.86
Sugar	3.8	5.66^a^ ± 0.00	0.9	0.77	6.39 ± 0.00	0.81
Salt	3.4	11.42^ac^ ± 4.43	2.6	0.86	6.31 ± 0.37	0.76

^1^ Different letters in the same column differ (*P* < 0.05)

^2^The shape parameter (n) indicates the shape of the curve with a value n > 1 forming shoulders and being convex, n < 1 forming tails and being concave, and n = 1 being linear.

The comparison of all feed additives in terms of active ingredient and pH value is shown in Table 5. In general, the additives containing phosphoric acid were more effective at reducing virus concentration. The only additive containing 2-hydroxy-4-methylthiobutanoic acid (Activate DA) was the most effective in reducing virus concentration. There were differences in the pH values of the feed samples after the addition of additives.

**Table 5:** Composition and properties of the feed additives used.

Company	Additive Name	Ingredients	pH^1^	Delta^1^ Value (days)
Novus	Activate DA	2-hydroxy-4-methylthio butanoic acid, fumaric and benzoic acid	5.50^c^ ± 0.03	0.81^a^ ± 0.52
Kemin	KEM-GEST	phosphoric, fumaric, lactic, and citric acids	5.74^b^ ± 0.03	3.28^a^ ± 2.05
Sugar	Sugar	Sucrose	5.88^e^ ± 0.03	5.66^a^ ± 0.00
AgriNutrition	Acid Booster	phosphoric, citric, and lactic acids	5.84^a^ ± 0.03	7.24^a^ ± 3.71
Salt	Salt	sodium chloride	5.84^a^ ± 0.02	11.42^ac^ ± 4.43
Nutriad	Ultracid P	orthophosphoric, citric, fumaric, and malic acids	5.73^b^ ± 0.01	13.00^ac^ ± 3.41
Control	None	no additive	5.82^a^ ± 0.02	17.23^bc^ ± 0.78

^1^ Different letters in the same column differ (*P* < 0.05)

## Discussion

Feed was suggested as a vehicle of transmission of PEDV when the first Canadian swine farm tested positive for the virus [9]. A follow-up epidemiological investigation suggested that a batch of spray-dried porcine plasma (SDPP) used in swine nursery feed was the potential source of PEDV infection. In a bioassay experiment, a contaminated SDPP sample was able to infect pigs, however, when added to the complete feed no additional pigs were infected. This was potentially caused by a dilution effect or an extended time period between virus contamination and bioassay [9]. A similar conclusion that feed or feed ingredients could be spreading PEDV between farms was made during an investigation of a PEDV outbreak in Ohio. In this situation, virus was detected in the feed through RT-PCR on a farm with infected sows. In a follow up bioassay, none of the pigs developed an infection after consuming the contaminated feed [7]. In this study, the source of feed contamination was not found, although the RT-PCR test was positive for PEDV. Dee et al. [10] showed that feed experimentally contaminated with PEDV RNA could cause an active infection in pigs when feed was consumed via natural feeding behavior. The collection of references indicates that if complete feed happens to be contaminated with PEDV, it is possible for the contaminated feed to cause an active infection on a farm. In summary, these results suggested that additional research is needed to evaluate potential mitigation strategies to control transmission of PEDV through feed processing methods.

The survival of PEDV in feed that was thermally treated at 120 to 145°C for 0 to 30 minutes was first evaluated. Previous research has focused on the survival of PEDV at temperatures below 80°C. One study found that a cell culture-adapted strain of PEDV was moderately stable at 50°C for 30 min, with only a reduction of 0.4 log_10_ PFU/mL at 50°C when compared to the control [1]. A further investigation indicated that treating PEDV at temperatures between 60 to 80°C for 30 min caused a complete loss of infectivity, but the log reduction in virus concentration was not reported [1]. In another study, the survival of a wild type strain of PEDV in fecal samples required only 10 min of exposure at 71°C to be inactivated to the extent that it was not able to cause infection in live pigs [27]. This variability in virus inactivation could be possibly due to differences in moisture content between the different samples tested. The moisture content will ultimately have an impact on virus survivability during heat treatment.

Although the exposure times and temperatures varied in these studies, their results correspond with previous data indicating that PEDV is a low thermally stable virus [4]. Our hypothesis was that the thermal treatment of feed would reduce the survival of PEDV in swine feed. This hypothesis was confirmed by our experimental results which demonstrated that a 3 log (99.9%) reduction of the PEDV concentration is achieved within 25 min of exposure at 120°C. These results also highlight the finding that temperatures over 130°C don’t increase the inactivation of PEDV. This is an important observation because excessive heating of high protein feed ingredients results in reductions of amino acid digestibility through Maillard reactions [28]. In addition to the log reduction of PEDV in feed at high temperatures, the production of a shoulder in the inactivation curve produced by the Weibull model also help determine PEDV survival at high temperatures. The observed shouldering phenomenon observed in the inactivation curves may indicate that virus is resistant to a certain temperature-time level combination and a threshold needs to be surpassed to see a significant level of inactivation. Another possible reason for this shoulder in the curve could be the possibility that the feed was cooled when the room temperature inoculum was added. This would mean the first few min the feed was placed back in the oven was spent heating back up to oven temperature, which could explain the plateau in virus concentration. Overall, the results from our experiment confirm that the concentration of PEDV in complete feed can be reduced through the application of heat.

When exploring non-thermal treatment processes to reduce PEDV in feed, an eBeam irradiation dose of 50 kGy was found to be effective in reducing virus concentration by 3 log (99.9%). Currently, the Food and Drug Administration has stipulated an upper dose limit of 50 kGy of ionizing irradiation doses in animal feeds [19]. The use of eBeam processing of animal feeds and diets has been shown to be an effective process for inactivating pathogens in the US [16,17]. Animal feed and bags (weighing about 20 kg) are routinely treated using eBeam processing. Process controls are in place to measure the maximum and minimum doses in these sample bags to meet customer needs and adhere to regulatory limits. Though we have shown that eBeam treatment is effective in reducing the concentration of PEDV in swine feed, follow-up studies are needed to verify the results seen in this study with different types of feed. Our experimental data, suggesting that a target dose of 50 kGy will achieve approximately 3 log_10_ reduction in PEDV, are important in that it highlights the criticality of ensuring low viral bioburden during feed formulation by adhering to Good Manufacturing Processes. These results suggest that if the initial PEDV bioburden is greater than 4 log_10_ TCID_50_, the FDA mandated 50 kGy dose upper limit may not be effective in complete inactivation of virus in these feeds. Furthermore, if the maximum titers of PEDV in commercial swine feed are known, then the dose can be calibrated to theoretically achieve the total elimination of PEDV in commercial swine feed. In addition to more research investigating maximum PEDV titers, it may also be necessary to investigate the use of irradiation on individual feed ingredients. Our experiment only measured the impact of irradiation on complete swine feed, and so it cannot be confirmed if the same results would be seen in individual feed ingredients.

Finally, we evaluated the survival of PEDV in feed when various feed additives were included at manufacturers’ recommended doses. The additives experiment was only performed at room temperature. On a swine farm, feed can be stored at a variety of temperatures based on season, barn layout, bagged feed vs. bulk feed, barn temperature, and other variables. Room temperature was used in this experiment as a feasible average temperature, though it may not accurately apply to every situation. Feed is suggested to be stored in a cool, dry place and so it is likely some swine feed will be stored at temperatures below the approximately 25°C tested in this experiment.

The addition of organic acid feed additives in the diet has been shown to increase nutrient digestibility by lowering pH and modifying gut microflora, which ultimately improve growth performance and provide an alternative to antibiotic use in weaned pig diets [29]. Organic acids have been used to reduce the prevalence of bacterial pathogens such as Salmonella in poultry feed [21]. In an additional experiment, the antimicrobial product SalCURB, which is used to control *Salmonella* in feed, was tested for its ability to decrease the presence of viable PEDV in feed. In this experiment, a bioassay showed that the feed treated with the SalCURB product was not able to cause an infection in naïve pigs [30]. A proposed method for how the SalCURB inactivated the PEDV was not reported. Differences in PEDV survival in feed has not only been observed in the presence of additives, but also in the presence of different feed ingredients. In an experiment testing 18 different feed ingredients, it was determined that the survival of PEDV in these ingredients was ingredient specific with an extended survival being observed in soybean meal [31]. This indicates that variation as small as changes in ingredients could change virus survival in feed.

It has been reported that PEDV is stable at a pH of 6.5 to 7.5 at a temperature of 37°C [32]. Because intestinal viruses must survive the acidic conditions of the stomach, they are generally more resistant to acidic pH. With this being said a change in pH outside of ideal conditions for an extended period could cause an increase in virus inactivation. A previous review reported that the pH of the diet was decreased from an average pH of 5.96 to 4.71 when organic acids (fumaric, propionic, formic, citric, sodium citrate, hydrochloric, sodium fumaric, or malic acid) were added to feed [33]. This pH decrease of the diet was hypothesized to decrease the survival of PEDV in the presence of additives. The results from our study did not necessarily support this hypothesis. Activate DA and KEM-GEST were the most effective in reducing virus survivability and also had the most acidic pH values when added to feed, which confirm the original hypothesis. However, Ultracid P did not result in a different diet pH compared with KEM-GEST, yet it had the highest delta value of all additives evaluated. In addition to this, the feed sample would require a type of liquid to be added to the feed for pH to even play a role, as a dry substrate will not have a pH. In the experiment, 1mL of virus media was added to the sample and mixed. This means it could be possible for the pH of the media to change because of the additive when the virus was initially added. If this single change were to explain the differences in survival for our experiment, there would be a more prominent pattern between the pH of the feed solution and the delta value observed. With a lack of a clear pattern between pH and virus survival, and in some cases no difference between the pH of the solution with the control and with some of the additives tested, there is another aspect causing the changes in virus survival between the feed additives used.

There are other components of feed additives that have the capability to reduce PEDV survivability, as suggested by the inability to explain the decrease in PEDV survivability due to change in pH alone. We hypothesized that specific active ingredients play a role in differences in virus survivability among additives. Many of the less effective additives contained citric acid and lactic acid as active ingredients. It has been previously shown that citric and lactic acids only have moderate antimicrobial activity because they are solely effective at a low pH in the environment [34]. Because most swine feed samples have a neutral pH, it is not surprising that additives containing citric acid as an active ingredient may not have been as effective in inactivating PEDV. In contrast, fumaric acid has been shown to be a very effective antimicrobial, and is extremely effective in reducing the survivability of *E*. *Coli* [35]. No studies have been published to show that fumaric acid is an effective anti-viral compound, but its presence in KEM-GEST, which resulted in the second lowest PEDV survivability in our study, implies it may also be an effective ingredient in reducing survivability of PEDV. The active ingredient of Activate DA, the most effective additive in our study, is 2-hydroxy-4-methylthiobutanoic acid. This ingredient has been researched exclusively as a cost effective supplement in low methionine diets for swine [36]. Although it has not been approved for use as an antimicrobial or antiviral agent, the results from our study suggest it could have potential in reducing virus survivability. Overall, careful selection of additives containing the most effective active ingredients is necessary for achieving reduced PEDV survivability in complete swine feeds. Further research is needed to confirm the relative effectiveness of each active ingredient and how they may interact with each other.

One limitation in this experiment is the unknown variable of water activity. By adding liquid media to the samples the moisture content was already dramatically increased. This increase in moisture potentially caused changes in the inactivation of the virus when exposed to heat that may not be observed in the low moisture conditions of animal feed. It has been demonstrated that PEDV inactivation using heat treatments and changes in pH differs in liquid media compared with a dried plasma product [37]. Because our experiments investigated both change in pH through an additive and change in heat treatment, our increased moisture from inoculating the sample could cause different inactivation kinetics than what would be seen in dry feed without media. In addition to this, the humidity in the environment was not controlled and the water activity of the samples were not measured. Without measuring these variable there is no way to tell the role they played in the inactivation of PEDV in the feed samples. In addition to unknown water activity, this experiment also had the limitation of low starting virus titers. This made it impossible to reach the standard of complete inactivation (typically 7 log reduction) because the starting virus titers were only 4 logs.

Our results show that PEDV survivability can be reduced using eBeam processing or thermal processing at temperatures greater than 130°C. The use of some feed additives was also effective in reducing virus levels, but future investigations are needed to determine the prevalence and maximum titers of PEDV in commercial swine feeds to determine the most appropriate treatment parameters (time and temperature of thermal processing, dose of eBeam processing, and feed additive selected) to be most effective in inactivating PEDV.

## Conclusions

Feed has been suggested as a possible vehicle of PEDV transmission. This has created a need to evaluate new and existing feed processing methods to reduce PEDV survivability in feed. Results from this study have shown that both thermal (130°C for at least 15 min) and non-thermal (50 kGy eBeam dose) feed processing technologies can eliminate 99.9% of PEDV infectivity when working with low virus titers (6.8x10^3^ TCID_50_/mL). Additionally, the survivability of PEDV can be reduced by the use of selected acidifiers and organic acids in swine feeds.
